# Concerning the Food We Eat

**Published:** 1887-12

**Authors:** Dumont C. Dake

**Affiliations:** 304 Fifth Ave., N. Y.


### CONCERNING THE FOOD WE EAT.

BY DUMONT C. DAKE, M.D., 304 FIFTH AVE., N. Y.

We have, in the different varieties of food we eat, the phosphates, the nitrates, and the carbonates. The first makes *brain*; the second makes *bone* and *muscle*; the last makes *fat.*

By analysis of the *leading* articles of *food*, we have the following results:

| | Phos., parts. | Nit., parts. | Carb., parts. |
|---|---|---|---|
| Beef gives | 5 | 15 | 20 |
| Mutton | 3 | 12 | 40 |
| Lamb | 3 | 12 | 35 |
| Pork | 1 | 10 | 50 |
| Veal | 4 | 16 | 16 |
| Salmon | 7 | 20 | 10 |
| Oysters | 2 | 10 | 10 |
| Codfish | 6 | 14 | 5 |
| Eggs | 5 | 16 | 0 |
| Wheat | 2 | 15 | 69 |
| Rye | 2 | 12 | 72 |
| Corn | 1 | 12 | 72 |
| Buckwheat | 1 | 8 | 15 |
| Barley | 3 | 17 | 10 |
| Oats | 3 | 17 | 66 |
| Beans | 4 | 24 | 57 |
| Peas | 3 | 22 | 60 |
| Rice | ½ | 6 | 80 |
| Potatoes | ½ | 1 | 22 |
| Turnips | ½ | 1 | 21 |
| Cabbage | 3 | 20 | 46 |
| Butter | 0 | 0 | 100 |

By the last item, one may see he can eat a firkin of butter and never get a thought. It, however, answers a good purpose, and supplies plenty of carbon. When brain force is required, brain food should be eaten. When physical labor is to be performed, the food that makes nerve and muscle is to be preferred.

In cold weather, food that supplies carbon should be taken, but less in hot weather. During April and May, and also in July and August, but little animal food should be eaten. A mixed diet is best, neither drawing nourishment entirely from the animal or vegetable kingdom.

Persons with small lungs demand more meat as a diet than those with large ones. Meat containing a large supply of nitrogen which is so essential to human existence.

Children under ten years of age should be allowed to eat but little or no meat. We consider pork to be injurious to the human system, having very little nutrition as compared with other articles of diet.

Drinking, while eating, is injurious to the health. It dilutes the gastric juices of the stomach, brings on dyspepsia, liver complaint and other diseases. Animals finish their meal, and then drink. Humans often eat by mouthfuls, washing the same down many times during *one meal* with liquids.

In its passage downward the food meets with, at least, five different secreted fluids, namely, *saliva* in the cavity of the mouth, the *gastric juice* in the stomach and the *intestinal juice*, with *pancreatic juice* and the *bile*, discharged into the cavity of the small intestine. About four ounces of the saliva which is of an alkaline reaction passes down with each meal we eat, this with the gastric juice of the sotmach, which is an acid reaction, digests the food assisted by the powerful action of the pneumogastric nerves. It is a great mistake to drink strong mineral waters also bicarbonate of soda for dyspepsia.

It gives temporary relief only but increases the flow of the gastric juices and the stomach becomes more acid.

We agree with the distinguished English Physician who says, "*The Chemistry of Food is the Coming Physician.*"

"*Eat to live*, not live to eat."

